# The association of thrombophilia in women with severe obstetric complications

**DOI:** 10.25122/jml-2022-0182

**Published:** 2022-10

**Authors:** Faisal Mousa Alzahrani, Abdulaziz Al-Mulhim, Saeed Sattar Shaikh, Maryam Ahmed Aldossary, Ahmed Aldarmahi, Yaser Alnaam, Lamiaa Hammad Al-Jamea, Thekra AL-Maqati, Elmoeiz Elnagi, Fathelrahman Mahdi Hassan, Muzaheed

**Affiliations:** 1 Department of Clinical Laboratory Sciences, College of Applied Medical Sciences, Imam Abdulrahman Bin Faisal University, Dammam, Saudi Arabia; 2 Department of Obstetrics and Gynecology, College of Medicine, Imam Abdulrahman Bin Faisal University, Dammam, Saudi Arabia; 3 Department of Basic Science, College of Science and Health Professions, King Saud Bin Abdulaziz University for Health Sciences, Jeddah, Saudi Arabia; 4 Department of Clinical Laboratory Sciences, Prince Sultan Military College of Health Science, Dhahran, Saudi Arabia; 5 Department of Hematology and Immunohematology, College of Medical Laboratory Sciences, Sudan University of Science and Technology, Khartoum, Sudan

**Keywords:** thrombophilia, pregnancy, obstetrics complications, Saudi Arabia

## Abstract

Thrombophilia, where multiple genetic and acquired risk factors interact synergistically, are associated with thrombosis and pregnancy-related complications. Despite being studied profusely, an inconsistent association exists between thrombophilia and pregnancy complications. Between 2018 and 2020, ninety-three women with pregnancy complications were enrolled in the study. Twenty-five healthy pregnant women without pregnancy complications reported to the same hospital were also recruited as controls. Blood samples were tested for homocysteine, coagulation studies, and molecular diagnosis included FVL, PTH and MTHFR genes amplified using PCR strip assay (Vienna Lab Diagnostics, Austria). Other thrombophilia screening, including testing for AT, PC, and LA, were done by chromogenic assays (Dade Diagnostica, Munich, Germany). Homocysteine level was determined by fluorescence polarization immunoassay technology (Axsym, Abbot company, Germany). Overall, 29.03% of women with pregnancy complications had thrombophilia relative to 16% in the control group. However, the difference between the case and control groups did not reach a significant level (p=0.1175). Additionally, combined thrombophilia was more prevalent among cases (10.75%) than in the control group (4%). However, the difference did not reach statistical significance (p=0.1046). Our study demonstrated that the frequency of thrombophilia among healthy women was 16%, and among women with pregnancy-related complications, 29%. Relative to control, all measured thrombophilia markers were more frequent in women with pregnancy-related complications except for LA. Including all the studies on the Saudi population in a meta-analysis study could reveal more information about thrombophilia and pregnancy-related complications in our population.

## INTRODUCTION

Severe preeclampsia, placental abruption, intrauterine growth restriction (IUGR), stillbirth and venous thromboembolism (VTE) contribute significantly to maternal morbidity and mortality. The risk of these severe pregnancy-related complications is higher in women with disorders associated with a persistent hypercoagulability state that predisposes them to thrombosis, known as thrombophilia [1]. Although there is no universally accepted definition of thrombophilia, the term mainly refers to haemostatic disorders detected in the laboratory, which lead to thromboembolic pathologies. Multiple genetic and acquired risk factors that interact synergistically are associated with aetiologies of thromboembolism which, when disturbing the placental vascularization, result in inadequate uteroplacental circulation associated with pregnancy complications [2]. The heritable thrombophilia includes a deficiency in the natural anticoagulants, including anti-thrombin (AT), protein C (PC), and protein S (PS), and the presence of specific variants affecting coagulation factors, most commonly factor V Leiden (FVL; p.Arg506Gln), and prothrombin (PTH; G20210A) variants. The presence of anti-phospholipid antibodies, including anti-cardiolipin antibodies (aCLA), anti-β2 glycoprotein I (anti-β2GPI) and lupus anticoagulant (LA) are also associated with the acquired form of thrombophilia. Furthermore, homocysteinemia caused by either variant affecting methylenetetrahydrofolate reductase enzyme (MTHFR; p.Cys677Thr) or acquired elevation in homocysteine level had been associated with hypercoagulability [3].

An enormous number of studies investigated thrombophilia and pregnancy-related complications and agreed that there is an increased frequency of thrombophilia in women with pregnancy complications. However, a variable degree of association between each thrombophilia and type of pregnancy complications was observed [3].

The observed heterogeneity between different types of thrombophilia, different types of pregnancy-related complications, and inconsistency in the magnitude of the risk is significant. A possible explanation for such discrepancy is the multi-factorial nature of thrombophilia, differences in the definition of pregnancy complication across studies, and different sensitivity and specificity of the laboratory methods used in testing for thrombophilia [3, 4]. Equally, genetic variations between ethnic populations studied could explain part of the observed heterogeneity. Considering the high level of consanguineous marriages in Saudi Arabia [4] and the difference observed in the frequency of thrombophilia markers in VTE Saudis patients compared to what is illustrated in the literature suggests a distinctive pattern of association between thrombophilia and pregnancy-related complications in the Saudi population [5]. A small number of thrombophilia studies in the Saudi population were carried out; however, they mostly focused on heritable thrombophilia [5–8]. Additionally, from the ones that focused on thrombophilia in women with recurrent pregnancy loss, none highlighted the frequency based on the placental dysfunction disorders. Because of the insufficient data in our country, this study aimed to examine the frequency of thrombophilia in Saudi women with severe obstetric complications in different placental dysfunction disorders compared to a control group of healthy pregnant women without obstetric complications.

## Material and Methods

A case-control study was conducted at King Fahd Teaching Hospital of the University, Alkhobar, Saudi Arabia, from January 2018 to January 2020. Ninety-three women with pregnancy complications, namely preeclampsia, placental abruption, IUGR, stillbirth and VTE, were enrolled in the study. In addition, twenty-five healthy pregnant women without pregnancy complications reported to the same hospital were recruited as controls. Written informed consent was obtained from each participant.

Blood samples were collected in a plain 3.2% trisodium citrate tube by an evacuated tube system. The serum tested for homocysteine was prepared by incubating the tube at 37℃ for 1–2 hours, then centrifuged at 3000 rpm for 10 minutes. The serum was separated, divided into small plastic aliquots of one ml each and frozen at -80℃. Immediately before performing the tests, the serum was thawed rapidly at 37℃ in a water bath for 10 minutes. Citrated plasma used for coagulation studies was prepared by centrifugation for 10 minutes at 3000 rpm immediately after blood collection.

The molecular diagnosis included FVL, PTH and MTHFR genes, amplified using PCR strip assay (Vienna Lab Diagnostics, Austria). QIAamp DNA Blood Mini Kit was used to extract and purify the DNA from samples. Other thrombophilia, including testing for AT, PC, and LA, were done by chromogenic assays (Dade Diagnostica, Munich, Germany). Finally, the homocysteine level was determined by fluorescence polarization immunoassay technology (Axsym, Abbot company, Germany).

### Statistical analysis

SPSS software (version 24) was used for all statistical calculations. The association between clinical presentation and thrombophilia was assessed using Fisher's exact test. A p-value less than 0.05 was considered significant.

## Results

### Study subjects

This study evaluated the presence of thrombophilia in 93 pregnant women with different pregnancy complications and 25 healthy pregnant women with a normal obstetric history (Table 1). Thrombophilia evaluation included the molecular diagnosis of FVL (R506Q), PTH (G20210A) and MTHFR (C677T) variants (Table 2), measuring the level of AT and PC, and detecting the presence of LA (Table 3). Individuals were considered to have thrombophilia when one or more of the following was detected: homozygous or heterozygous of FVL variant (R506Q), homozygous or heterozygous of PTH variant, homozygous MTHFR variant, low level of AT, low level of PC, and detection of LA.

**Table 1:** The number of women with pregnancy-related complications and controls included in the study.

	No. of cases
**Case group**	**93**
Preeclampsia	25
Placental abruption	23
IUGR	19
Stillbirth	20
VTE	6
**Control group**	**25**

**Table 2:** Frequency of FVL, PTH and MTHFR variants among cases with pregnancy complications and control.

	MTHFR^	PTH*	FVL*
**Case (n=93)**	**2.15**	**5.38**	**3.23**
Preeclampsia	4	4	0
Placental abruption	0	0	4.35
IUGR	0	10.53	5.26
Stillbirth	5	5	5
VTE	0	16.67	0
**Control (n=25)**	**0**	**4**	**0**

^ – homologous; * – heterozygous.

**Table 3:** Frequency of AT, PC, LA among cases with pregnancy complications and control.

	AT	PC	LA
**Case (n=93)**	**15.05**	**12.90**	**1.08**
Preeclampsia	8	12	0
Placental abruption	30.43	26.09	0
IUGR	15.79	5.26	0
Stillbirth	0	0	0
VTE	33.33	33.33	16.67
**Control (n=25)**	**4**	**4**	**8**

### Frequency of overall thrombophilia in case versus the control group

**Figure 1:**
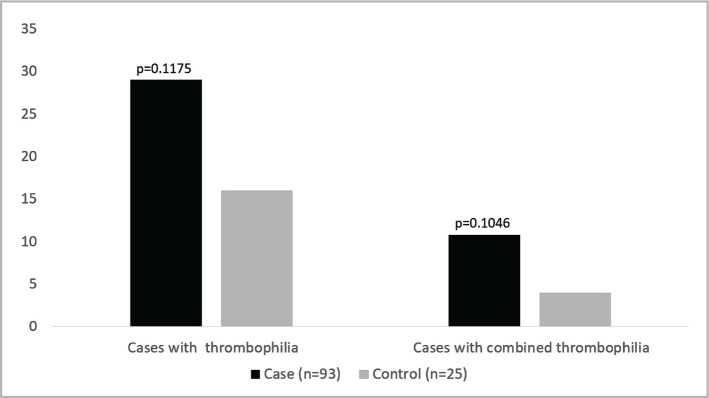
Frequency of thrombophilia and combined thrombophilia in cases with pregnancy complications than in controls.

29.03% of women with pregnancy complications had thrombophilia relative to 16% in the control group. However, the difference between the case and control groups did not reach a significant level (p=0.1175). Additionally, combined thrombophilia was more prevalent among the case (10.75%) than the control (4%) group. However, the difference did not reach statistical significance (p=0.1046) (Figure 1).

### Frequency of overall thrombophilia in different obstetric complications versus the control group

**Figure 2:**
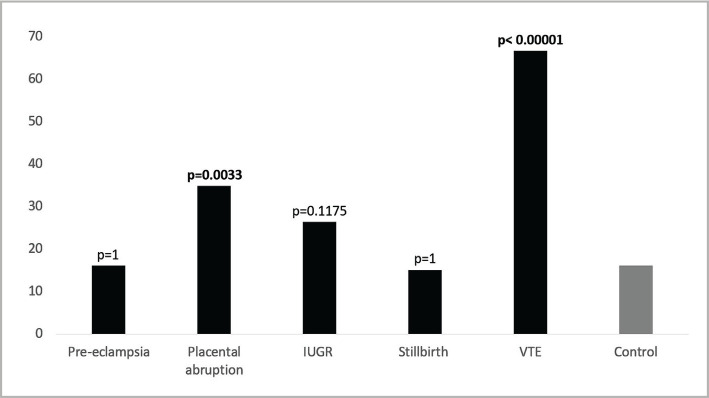
Frequency of thrombophilia in different obstetric complications and control group.

The percentage of overall thrombophilia in different obstetric complications and the control group revealed that thrombophilia was higher among cases in every obstetric complication relative to control except for women with preeclampsia and stillbirth (Figure 2). Nevertheless, the difference in the frequency was significantly higher only in women with placental abruption (p=0.0033) and VTE (p<0.00001).

### Frequency of different thrombophilia types among cases and control groups

**Figure 3:**
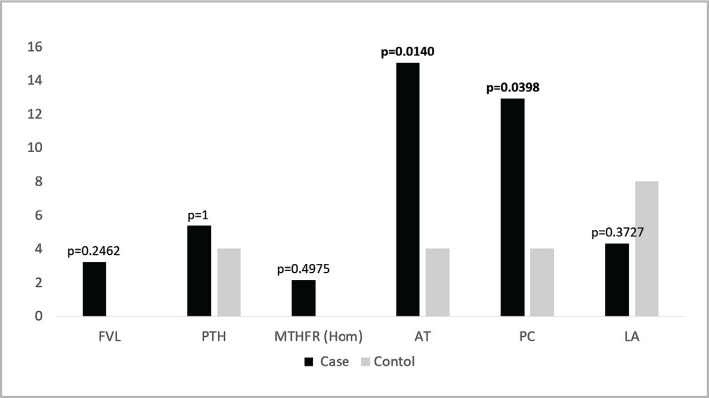
Frequency of different types of thrombophilia among cases and control.

Each type of thrombophilia was more prevalent in cases than the control group except for LA (Figure 3). From all evaluated thrombophilia, the FVL variant and homozygous MTHFR variant were detected only in 5 cases. Noticeably, the frequency of AT and PC deficiency were more than double in cases relative to control, the differences reaching statistical significance (p=0.0140 and 0.0398, respectively).

### Different thrombophilia among different obstetric complications versus control

#### A. Thrombophilia molecular diagnosis

The thrombophilia molecular diagnosis in this study included FVL, PTH and MTHFR variants. The frequency of these thrombophilic variants in the current cohort is presented in Table 2. These thrombophilia markers were highlighted in 10.75% of cases and 4% of control (p=0.1046). FVL variant was found in about 4–5% of women with placental abruption, IUGR, and stillbirth. Similarly, the homozygous MTHFR variant was found in 4–5% of women with preeclampsia and stillbirth. The frequency of the PTH variant varied between different complications, 11% IUGR, 5% stillbirth, and 17% VTE which were more prevalent compared to the control.

#### B. Thrombophilia haemostatic variables

The other thrombophilia evaluated in this study was the level of AT, PC, and the presence of LA, which was measured by either chromogenic or fluorescence-based assays.

Table 3 shows the frequency of measured thrombophilia variables in the case and control groups. Low AT and PC levels varied between pregnancy complications, but both were absent in women with stillbirth. Both AT and PC deficiency were present in about 30% of women with placental abruption and VTE. Furthermore, 8% of preeclampsia and 16% of IUGR had AT deficiency, compared to 12% of preeclampsia and 5% of IUGR who had PC deficiency. Positive LA, which was more frequent in cases than control, was found only in women with VTE.

## Discussion

Thrombophilia is known to vary according to the genetic background of the studied populations, which could explain part of the observed heterogeneity [2, 5]. Types of thrombophilia differ in different geographic regions [9]. A small number of publications mostly focused on the frequency of heritable thrombophilia in the Saudi population. Generally, the acquired thrombophilia markers are avoided due to variations in the laboratory tests used for their detection, and alteration in their level is associated with a normal physiological condition, *e.g*. pregnancy. These Saudi studies investigated the frequency in the general population [5, 7] or women with recurrent pregnancy loss [6, 8]. However, none of these studies included acquired thrombophilia markers nor looked at the frequency in different placental dysfunction disorders. We, therefore, aimed to contribute to the literature and fill this gap by examining the frequency of the most common heritable and acquired thrombophilia in Saudi women with pregnancy complications. In this case-control study, 93 Saudi women with pregnancy-related complications were compared to a control group of 25 healthy pregnant women. The pregnancy-related complications include preeclampsia, placental abruption, IUGR, stillbirth and VTE. Thrombophilia testing included the molecular diagnosis of FVL (R506Q), PTH (G20210A) and MTHFR (C677T) variants and measuring the level of AT, PC, and detecting the presence of LA.

The association between thrombophilia and placenta-mediated pregnancy complications makes some biological sense, yet in most publications, the association and the frequency of thrombophilia were inconsistent. In this study, more women with pregnancy complications had thrombophilia than control; however, the difference did not reach a significant level (29% and 16%, respectively, p=0.1175). The reported frequency of overall thrombophilia in women with pregnancy complications was lower than described in an early report, where 65% of women with a pregnancy complication had a form of heritable or acquired thrombophilia compared to 18% of women with a normal pregnancy [10]. Interestingly, this lower frequency could be related to a lower incidence of heritable thrombophilia documented in healthy Saudi subjects relative to other populations [7].

Comparing the frequency to other studies in a Saudi population, 11% of women had positive thrombophilia molecular markers and 4% of control reported in this study. This frequency is lower than one of the studies, which highlighted 72% of women with a history of recurrent pregnancy loss with inherited thrombophilia compared to 13% in control [6] but in agreement with a more recent one that reported hereditary thrombophilia in 23% of woman with recurrent thrombosis compared to 5% in the general population [8]. Combined thrombophilia was highlighted in about 11% of cases and 4% of the control group in the current study. A similar percentage of combined hereditary thrombophilia was observed among Saudi women with recurrent pregnancy loss but not in any of the controls [6].

In this study, the frequency of thrombophilia in every studied obstetric complication was higher relative to control except for subjects with preeclampsia and stillbirth. Conflicting results have been found in literature concerning thrombophilia and preeclampsia, despite the described association with ischemic placental lesions predisposing to hypercoagulability [11]. While some meta-analyses observed the associations [12, 13], others highlighted that there is no significant impact of thrombophilia on the risk of preeclampsia [14, 15]. Additionally, several studies reported an increased risk of preeclampsia for only certain thrombophilia markers, the PTH variant [12], the FVL variant [3], the homozygotes MTHFR variant [3], and anti-phospholipid antibodies [1]. The observed disagreements between outcomes could be explained by differences in ethnicity, study design, power, pooling of severity (mild, severe), and stage (early, late) of preeclampsia that likely resulted in an inaccurate evaluation of the precise impact of thrombophilic defects on preeclampsia. Stillbirth or, more generally, late pregnancy loss beyond 24 weeks gestation is associated in many studies with thrombophilia. It was found to be significantly increased in women with FVL, PTH, and anti-phospholipid antibodies, including LA, aCLA and anti-β2GPI antibodies but most strongly associated with PS deficiency [12, 16]. Not including PS level as one of the thrombophilia markers in this study and the small sample size could explain, at least partially, the absence of a significant increase in the frequency in women with stillbirth relative to control.

We also found that thrombophilia was more prevalent in cases than in control except for the presence of LA, detected in 8% of the control group compared to 1% of the case group. In a meta-analysis study, the incidence of anti-phospholipid antibodies, including LA and aCLA antibodies, in women with pregnancy complications was only 2% [12], which is near to what was found in this study. This high frequency of positive LA in the general population agrees with another study that includes a large population of Saudi women, which found LA in 5% of women with normal pregnancies [17]. On the other hand, the low frequency of LA among women with pregnancy complications relative to control was not an expected finding, and no compelling reason could justify this finding. However, aspects related to the transient nature of these antibodies, their association with infection or drugs, methods used for their detection and the cut-off value used could all have some impact.

In addition to LA, PTH was not significantly different between cases and control. Oppositely, from all evaluated thrombophilia, FVL and homozygous MTHFR variants were detected only in cases, and statistically, only the low AT and low PC were significantly different. It is reported that following the homozygous FVL variant, patients with AT deficiency, PC deficiency, and then heterozygous FVL were respectively associated with the highest risk of VTE [18]. Regarding the PTH, although a study on the Saudi population highlights its involvement in increasing recurrent miscarriage incidence [6], a lack of significant association was observed with any adverse pregnancy-related outcomes in several studies [3, 15]. Similar to other thrombophilia, an inconsistent association between the MTHFR variant and pregnancy complication were described [3, 6, 8, 12]. One possible explanation is the zygosity state for the MTHFR variant, as some studies include both zygosities, while others restrict the analysis to the homozygous transition only. An additional explanation is the possible confounding effects of the folic acid supplement that a pregnant woman usually takes on homocysteine levels [19].

In addition, Safdarian and her team found that inherited thrombophilia in cases of *in vitro* fertilization (IVF) procedures is more prevalent in women with recurrent IVF failure than in healthy women. They indicated that the presence of factor V Leiden mutation and the homozygote form of MTHFR mutation were risk factors for recurrent IVF failure [20]. Establishing an *in vitro* model to study the effect of thrombophilia within the female reproductive tract could lead to more insightful interactions [21].

Multiple reasons could justify the variation in the result between this study and others and among other studies themselves—first, the limited marker of thrombophilia measured. In this study, the level of PS, homocysteine or other anti-phospholipid antibodies, other variants in the FV, and promoter variant in the PAI-1, among others, were not included. Second, the small number of study subjects and the lack of information regarding the consanguinity, history of gestational and thrombosis. Equally important, the variation of the laboratory tests used between the studies and the severity of the pregnancy complication could explain some of the heterogeneity.

The frequency of thrombophilia markers was 16% in women with preeclampsia, 35% placental abruption, 26% IUGR, 15% stillbirth and 67% VTE. Placental abruption and VTE were significantly more frequent in women with thrombophilia. Including all the studies on the Saudi population in a meta-analysis study could reveal more information about thrombophilia and pregnancy-related complications in our population. Also, it could aid in developing tiers of screening analysis tests tailored for Saudi women with a history of pregnancy-related complications based on the overall frequency. Interestingly, Saour et al. established a collaborative Saudi national registry in 2009 for the heritable thrombophilia variants and reported the preliminary data for 900 healthy subjects for the prevalence of five prothrombotic gene variants [7].

Robertson et al. (2006) conducted a systematic review, including 79 studies, to determine the risk of VTE and adverse pregnancy outcomes in pregnant women with thrombophilia [12]. The result indicates that all heritable thrombophilia were associated with pregnancy complications but in variable patterns of association relative to the type of complication. While FVL was associated with the highest risk as it was significantly associated with all pregnancy complications except IUGR, homozygous MTHFR was only significantly associated with preeclampsia. Another nested case-cohort study of pregnant women that included 2,032 cases and 1,851 random controls supports an increased risk of pregnancy complications with FVL, including severe preeclampsia, IUGR and placental abruption [3]. Oppositely, Rodger et al. (2010), using a meta-analysis of prospective cohort studies that included more than 20,000 women, highlighted a significant association of FVL with only late pregnancy loss but not with other complications [2].

Regarding acquired thrombophilia, Robertson et al. (2006) also evaluated this type of thrombophilia and showed that LA was associated with a higher risk of early pregnancy loss, while aCLA was associated with all pregnancy complications except placental abruption. Another meta-analysis that targeted women without autoimmune diseases to evaluate the association between the presence of aCLA and LA and recurrent fetal loss highlighted that aCLA was associated with early recurrent fetal loss, and both aCLA and LA were associated with late fetal loss [22]. Comparable findings were highlighted by a more recent systematic review with a similar aim [1].

Our study does not have data on whether some participants were treated with low molecular weight heparins (LMWH). Since treatment with low molecular weight, heparins prevent adverse pregnancy outcomes in patients with isolated or combined hereditary thrombophilias [23, 24], and this kind of data is lacking, we acknowledge this as a study limitation. Also, a larger sample size on a wide geographical area is required to best accomplish such studies.

## Conclusion

Our study demonstrated that the frequency of thrombophilia among healthy women was 16%, and among women with pregnancy-related complications, 29%. Relative to control, all measured thrombophilia markers were more frequent in women with pregnancy-related complications except the LA. Including all the studies on the Saudi population in a meta-analysis study could reveal more information about thrombophilia and pregnancy-related complications in our population.

## Data Availability

The data used to support the findings of this study are included in the article.
